# The Evolution of Artificial Intelligence in Antibody Design: From Structure-Based Engineering to Generative Models

**DOI:** 10.3390/antib15050081

**Published:** 2026-09-02

**Authors:** Ida Szataniak, Kacper Packi

**Affiliations:** 1 IMPAKT—Interdisciplinary Student Research Group of Immunology, Allergology, Parasitology and Molecular Medicine, Department of Medical Sciences, Władysław Biegański Collegium Medicum, Jan Długosz University in Częstochowa, 42-200 Częstochowa, Poland; k.packi@ujd.edu.pl; 2 Department of Immunoparasitology and Molecular Allergology, Jan Długosz University in Częstochowa, 42-200 Częstochowa, Poland; 3 AllerGen Center of Personalized Medicine, 97-300 Piotrków Trybunalski, Poland

**Keywords:** artificial intelligence, antibody engineering, computational antibody design, deep learning, protein language models, generative artificial intelligence, antibody structure prediction, therapeutic antibodies, computational biology

## Abstract

**Background/Objectives**: Artificial intelligence (AI) has transformed computational antibody engineering by enabling accurate prediction of antibody structures, rational optimization of therapeutic properties, and de novo antibody design. Recent advances in deep learning, protein language models, and generative AI have fundamentally changed the way antibodies are discovered and engineered. This review aims to present the historical evolution of computational antibody engineering, from early structure-based design strategies to modern AI-driven approaches, while highlighting the major computational tools, publicly available databases, current limitations, and future directions of the field. **Methods**: A comprehensive narrative review of the literature was conducted using PubMed, Scopus, Web of Science, and Google Scholar. Original research articles, methodological studies, and review papers published between 1985 and 2026 were evaluated. Publications were selected according to their scientific relevance, methodological quality, and contribution to the historical development of computational antibody engineering. **Results**: The review describes the progression of antibody engineering from phage display and structure-based computational methods to machine learning, deep learning, protein language models, and generative artificial intelligence. It summarizes key public databases supporting antibody research, discusses advances in antibody structure prediction and developability assessment, and reviews recent generative models capable of designing antibody sequences and structures. Current challenges, including limited experimental validation, dataset bias, prediction of highly flexible regions, model interpretability, and clinical translation, are also discussed. **Conclusions**: Artificial intelligence has fundamentally reshaped computational antibody engineering by integrating sequence, structural, and functional information into increasingly accurate predictive and generative frameworks. Although important challenges remain, recent developments indicate that AI-driven approaches will play an increasingly central role in the discovery and optimization of next-generation therapeutic antibodies.

## 1. Introduction

The continuous expansion of experimentally determined antibody structures stimulated the development of dedicated databases specifically designed to support computational antibody research [1,2,3,4]. Among these resources, the Structural Antibody Database (SAbDab) became one of the most comprehensive repositories by integrating antibody structures with standardized annotations, antigen information, affinity measurements, and experimental metadata [1]. Additional databases, including PyIgClassify, AbDb, Thera-SAbDab, the Observed Antibody Space (OAS), AB-Bind, and the Immune Epitope Database (IEDB), further expanded the availability of curated structural, sequence, mutational, and functional data for antibody engineering applications [2,3,4,5,6,7]. These resources substantially improved data accessibility and standardization, enabling increasingly sophisticated computational analyses of antibody structure, sequence diversity, antigen recognition, and mutational effects [1,2,3,4,5,6,7].

The availability of large, well-curated datasets also facilitated the advancement of standardized antibody numbering systems and structural classification schemes that enabled meaningful comparisons between antibodies with highly diverse sequences and structures [2,8,9,10]. One of the earliest and most influential concepts was the classification of complementarity-determining regions into canonical structures, demonstrating that most antibody hypervariable loops adopt a limited number of recurrent conformations despite extensive sequence diversity [8]. This observation provided the structural basis for predicting antibody loop conformations directly from sequence information and represented an important milestone in structure-based antibody modeling [8,9]. Subsequent numbering schemes, including the Kabat, Chothia, Martin, AHo, and IMGT systems, introduced standardized residue indexing approaches that greatly facilitated structural comparison, antibody annotation, and computational analysis [9,10]. These standardized frameworks became essential components of virtually all modern computational pipelines for antibody modeling and engineering [2,9,10].

The growing availability of experimentally validated structural and mutational datasets also enabled progress in computational methods capable of predicting the effects of amino acid substitutions on antibody stability and antigen binding [5,11]. Resources such as AB-Bind systematically collected experimentally measured changes in antibody–antigen binding affinity following mutations, providing valuable benchmark datasets for computational model development and validation [5]. At the same time, advances in rational protein engineering increasingly incorporated high-resolution three-dimensional structural information into the design process [12,13]. Structure-based computational approaches enabled researchers to identify key intermolecular interactions, evaluate energetic contributions of individual residues, and computationally prioritize mutations before experimental validation [11,12,13,14]. These methods significantly reduced the experimental burden associated with antibody optimization and improved the efficiency of rational engineering strategies [12,13,14]. Despite these important advances, classical computational antibody engineering remained largely dependent on experimentally determined structures and manually designed scoring functions [11,12,13,15]. Most early computational methods focused on predicting antibody–antigen interactions, estimating mutational effects on binding affinity, or assisting rational optimization of existing antibodies rather than generating entirely novel antibody sequences [10,11,15,16,17,18,19,20,21,22,23]. Computational tools primarily served as decision-support systems that complemented experimental antibody engineering instead of functioning as autonomous platforms for antibody design [10].

## 2. Aim

The aim of this review was to present the historical evolution of computational antibody engineering, from the earliest structure-based design strategies to contemporary artificial intelligence-driven approaches. Particular emphasis was placed on the transition from physics-based methods to machine learning, deep learning, protein language models, and generative artificial intelligence. In addition, the review summarizes major computational tools, publicly available antibody databases, and current challenges and future perspectives in AI-assisted antibody design.

## 3. Materials and Methods

This article presents a narrative review of the literature on computational antibody engineering and artificial intelligence applications in antibody design. The literature search covered publications published between 1985 and 2026 and was conducted from 14 April to 21 July 2026 using PubMed, Scopus, Web of Science, and Google Scholar.

The literature search was conducted iteratively, and search queries were progressively adapted to the individual topics covered by the review. The search strategy used different combinations of predefined keywords and Boolean operators (AND/OR), including “antibody engineering,” “antibody design,” “computational antibody design,” “artificial intelligence,” “machine learning,” “deep learning,” “antibody structure prediction,” “antibody modelling,” “antibody modeling,” “protein language models,” “generative artificial intelligence,” “generative AI,” “diffusion models,” “therapeutic antibodies,” “developability,” “affinity prediction,” and “affinity maturation.” Search terms were combined and adapted according to the thematic area under investigation and the characteristics of the individual database. Additional targeted searches were performed using the names of individual methods, models, databases, and resources discussed in the review to identify relevant methodological publications and subsequent applications. The principal database searches were further complemented by backward citation tracking of relevant publications.

Original research articles, methodological studies, and review articles published in English were considered eligible. Publications were included when they addressed one or more areas relevant to the scope of the review, including experimental antibody-engineering technologies that contributed to the development of computational approaches, antibody repertoire analysis and databases, structural modelling and prediction, machine learning and deep learning, protein language models, generative antibody design, affinity optimization, developability assessment, or other AI-assisted antibody-engineering applications. Particular consideration was given to original studies introducing influential computational methods or resources, studies providing experimental or quantitative validation of these approaches, and publications of historical importance to the development of the field. Review articles were used primarily to contextualize major methodological developments and to identify additional relevant primary studies.

Non-English publications, publications without sufficient methodological information, and studies outside the scope of computational or AI-assisted antibody engineering were excluded. Study selection was conducted iteratively and was guided by relevance to the predefined thematic scope of the review. Potentially relevant publications identified through the principal database searches, targeted searches, and citation tracking were evaluated for their relevance and contribution to the narrative synthesis. Reference lists of key publications were additionally examined to identify influential studies that were not retrieved through the principal database searches.

Approximately 110 publications were ultimately included in the review. Final inclusion was based on relevance to the thematic scope of the review, methodological contribution, scientific quality, historical significance, and contribution to understanding major developments in computational antibody engineering and AI-driven antibody design.

## 4. Results

### 4.1. Historical Evolution of Computational Antibody Engineering

#### 4.1.1. Phage Display and the Origins of In Vitro Antibody Engineering

The foundations of modern antibody engineering were established with the evolution of phage display, which enabled foreign peptides and later complete antibody fragments to be displayed on filamentous bacteriophages while maintaining a direct link between the displayed phenotype and its encoding genotype [24,25,26]. This innovation allowed functional antibody variable domains to be selected according to antigen-binding properties and demonstrated that rare high-affinity binders could be efficiently isolated from highly diverse libraries by affinity selection [24,25].

The implementation of combinatorial libraries of heavy- and light-chain variable regions markedly expanded the diversity available for antibody discovery and enabled the generation of antibodies without prior immunization [27]. Increasing library size further improved the probability of identifying high-affinity binders, establishing phage display as an effective platform for in vitro antibody selection [26,27,28].

Phage display subsequently emerged as an alternative to hybridoma technology by enabling direct isolation of antibodies from antibody-gene repertoires [27,29]. PCR amplification of rearranged variable-region genes greatly increased accessible sequence diversity and facilitated routine construction of large antibody libraries [26,29]. These libraries evolved into immune, naïve, semi-synthetic and synthetic formats, the latter allowing CDR composition and sequence diversity to be designed in silico before experimental synthesis [29,30].

#### 4.1.2. Yeast Surface Display in Antibody Discovery and Engineering

Yeast surface display emerged as a complementary platform for antibody discovery and engineering, enabling antibody fragments to be presented on the surface of Saccharomyces cerevisiae for combinatorial library screening [31]. In the classical system, proteins are displayed as C-terminal fusions to Aga2p, which is linked by disulfide bonds to the cell-wall-anchored Aga1p subunit [31]. Compared with bacterial expression systems, yeast provides protein-folding, secretory, and post-translational processing machinery resembling that of mammalian cells, facilitating the display of mammalian-derived antibody fragments [31,32,33].

A major advantage of yeast surface display is its compatibility with flow cytometry, which enables quantitative discrimination of clones according to binding affinity and dissociation kinetics using fluorescently labeled antigens [32,33]. The linkage between the displayed antibody phenotype and its encoding plasmid additionally allows binding properties to be characterized directly on the yeast surface without prior subcloning, expression, and purification [33]. These properties make yeast display particularly useful for directed evolution and affinity maturation, with mutagenic scFv libraries generated by error-prone PCR and DNA shuffling yielding variants with affinities as low as 48 fM and dissociation half-times exceeding five days [32,33].

Beyond affinity maturation, yeast display has been applied to antibody discovery from highly diverse libraries and to multiple antibody formats [32,33]. More than 50 fully human scFv clones were isolated from a nonimmune library exceeding 10^9^ variants using combined MACS and FACS selection, while Fab, single-chain Fab, IgG1 Fc, whole IgG, and camelid VHH domains have also been successfully displayed [32]. These applications demonstrate the versatility of yeast display as both a screening and antibody-engineering platform [32,33].

The technology has further been extended to fully synthetic in vitro nanobody discovery [34]. A yeast-displayed library designed from structurally characterized nanobodies contained at least 1 × 10^8^ unique full-length clones and combined initial MACS enrichment with subsequent FACS selection [34]. This strategy enabled rapid isolation of target- and conformation-selective nanobodies, including binders against human G-protein-coupled receptors [34]. Thus, yeast surface display complements phage display by combining eukaryotic protein processing with quantitative flow-cytometric screening and supporting the discovery and optimization of diverse antibody formats, including scFv and VHH molecules [31,32,33,34].

#### 4.1.3. Antibody Libraries and Affinity Maturation

Progress in antibody engineering was driven not only by increasing library diversity but also by improved control over library composition and selection conditions. Library quality became a major determinant of successful antibody discovery, while optimization of antigen concentration, washing conditions, temperature and counter-selection enabled the enrichment of antibodies with predefined binding characteristics and reduced off-target recognition [26,30,35,36].

Phage display also established an efficient platform for in vitro affinity maturation through iterative cycles of mutagenesis and antigen selection [25,29]. Random or targeted mutations introduced into antibody variable regions could be combined with progressively more stringent selection conditions to isolate variants with improved affinity and slower dissociation rates [25,26,29,36]. Recombinant affinity maturation produced antibodies with substantially higher affinities than their parental molecules, demonstrating that antibody sequences could be systematically optimized beyond their original binding properties [36].

The evolution in large naive, semi-synthetic and synthetic libraries further strengthened phage display as a versatile antibody-engineering platform [30,35]. Synthetic diversification enabled controlled exploration of sequence space, whereas expanded libraries generally increased both antibody specificity and average affinity [30]. Platforms such as HuCAL illustrated the potential of this strategy by generating antibodies with picomolar affinities, establishing phage display as a scalable, cost-effective and widely adopted technology for antibody discovery and engineering [30,35].

#### 4.1.4. Integration of Next-Generation Sequencing into Antibody Discovery

The introduction of next-generation sequencing (NGS) represented a major advance in antibody engineering by enabling millions of antibody sequences to be analyzed simultaneously and providing comprehensive insight into the diversity and dynamics of antibody libraries during selection [37,38,39]. Unlike conventional colony screening and Sanger sequencing, NGS identified both dominant and low-frequency variants, allowing antibody candidates to be selected based on enrichment patterns across successive rounds of biopanning rather than random clone selection [37,38].

Integration of NGS with phage display improved the identification of high-affinity antibodies and revealed functional variants that would have remained undetected using traditional screening methods [37,38,39]. Reconstruction of selected CDR combinations further enabled sequencing data to be converted into experimentally testable antibody clones, while analysis of rare variants demonstrated that sequence abundance alone did not necessarily predict functional quality [38,39].

A further methodological improvement was the preservation of native heavy- and light-chain pairing through microfluidic single-cell approaches [40]. Maintaining cognate VH–VL pairing generated libraries with higher sensitivity and specificity than randomly paired repertoires, reduced false-positive and false-negative discovery, and improved recovery of naturally occurring antigen-binding antibodies [40]. These advances transformed antibody discovery from limited clone-based screening into high-throughput interrogation of complete antibody repertoires [37,38,39,40].

#### 4.1.5. Emergence of Computational Antibody Engineering

The rapid expansion of antibody sequence and structural data laid the foundation for computational antibody engineering [10]. Early bioinformatic approaches focused on predicting antibody–antigen interactions, epitope recognition and mutational effects, but they were primarily intended to support the rational optimization of existing antibodies rather than generate novel binding specificities [10].

Standardized antibody numbering schemes, including Chothia, Martin and IMGT, together with consistent CDR definitions, enabled reliable structural comparison, annotation and computational analysis of antibody variable domains [10]. At the same time, classification of CDR conformations provided a structural framework for predicting loop geometry from sequence, although accurate modelling of highly variable CDR-H3 remained a major challenge [10].

These developments marked the transition from purely experimental antibody discovery toward structure-guided computational engineering. By integrating sequence information, structural knowledge and experimental datasets, computational methods became valuable tools for antibody modelling, affinity optimization and rational design, providing the conceptual basis for subsequent artificial intelligence-based approaches [10,30,37,39].

### 4.2. Antibody Repertoires, Immunogenomics and Public Databases

#### 4.2.1. High-Throughput Antibody Repertoire Sequencing

The development of high-throughput sequencing transformed antibody repertoire analysis into a quantitative approach for investigating adaptive immune responses and antibody diversity [41,42]. Millions of antibody sequences can be analyzed simultaneously, providing insights into clonal diversity, immune responses and antibodies with potential clinical relevance [5,41,43].

A major challenge in repertoire sequencing has been the preservation of native heavy- and light-chain pairing, as conventional approaches frequently analyzed VH and VL repertoires separately [41]. Single-cell emulsion-based sequencing addressed this limitation by enabling high-throughput recovery of paired VH–VL sequences, thereby improving reconstruction of complete antibody specificities and supporting analyses of public clonotypes and broadly neutralizing antibody-like features [41].

The quality of repertoire datasets also depends on library preparation and bioinformatic processing [42]. PCR-based workflows provide efficient and practical library construction, although extremely low RNA input can reduce clonal representation despite preserving the most abundant clones [42]. Standardized experimental protocols and computational pipelines are essential for generating reliable and comparable repertoire datasets [42].

#### 4.2.2. Computational Analysis of Antibody Repertoires

The increasing volume of repertoire-sequencing data required computational tools capable of transforming raw sequencing reads into annotated clonotypes [44,45]. Platforms such as MiXCR enabled automated identification of V(D)J gene usage, CDRs, clonotypes and somatic hypermutations while supporting high-throughput processing of large immunoglobulin datasets [45]. Standardized annotation systems, including IMGT, further facilitated integration of sequencing data with structural and functional antibody information across multiple databases [1,2,5,42,46,47].

Large-scale repertoire analyses revealed the coexistence of public clones shared among individuals and private clones unique to individual immune repertoires [41,47]. Machine-learning approaches, including sequence-kernel methods and support vector machines, successfully distinguished these repertoire classes, demonstrating that CDR3 sequence patterns were more informative than germline-gene usage or amino acid composition alone [47].

Network-based analyses provided an additional framework for studying repertoire organization by representing clonotypes as interconnected sequence-similarity networks [43]. These studies demonstrated that antibody repertoires exhibit reproducibility, robustness and redundancy despite extensive sequence diversity, supporting the application of network analysis to antibody discovery, synthetic repertoire design and personalized immunogenomics [43].

#### 4.2.3. Public Immune Repertoire Databases

The rapid expansion of antibody repertoire sequencing created a need for standardized public repositories capable of organizing, annotating and distributing immune repertoire data [5,44]. Resources such as the Observed Antibody Space (OAS) provide curated, IMGT-numbered antibody sequences together with standardized metadata, enabling large-scale comparative analyses of antibody repertoires across different organisms, immune states and experimental studies [5].

The Pan Immune Repertoire Database (PIRD) further extended these capabilities by integrating annotated B-cell and T-cell receptor repertoires with project metadata, visualization tools and interactive analytical functions [44]. In addition to serving as repositories, these databases provide reusable datasets for benchmarking computational methods, machine-learning applications and large-scale immunogenomic studies [44].

Public repertoire databases transformed fragmented sequencing datasets into standardized, searchable and interoperable resources that support antibody discovery, comparative repertoire analysis and the expansion of computational antibody-engineering methods [5,44].

#### 4.2.4. Structural Databases Supporting Computational Antibody Engineering

The growing number of experimentally determined antibody structures stimulated the development of dedicated structural databases supporting computational antibody engineering [1,4,48]. While the Protein Data Bank (PDB) remains the primary repository for biological macromolecular structures, antibody-specific resources provide standardized annotations and functionality tailored to antibody research [1,48].

SAbDab became one of the principal structural repositories by integrating antibody structures with experimental metadata, antigen information, binding affinities and standardized numbering schemes [1]. Complementary resources, including PyIgClassify and AbDb, further supported computational analyses by providing structural classification of CDR loops, processed antibody structures and multiple antibody-numbering systems [2,4].

Specialized databases subsequently expanded these capabilities for particular applications. Thera-SAbDab links therapeutic antibodies with experimentally determined structures, CoV-AbDab integrates structural and functional data for coronavirus-binding antibodies, and AB-Bind provides experimentally validated mutation-induced changes in antibody–antigen binding affinity for benchmarking computational prediction methods [3,7,46]. In parallel, the Immune Epitope Database (IEDB) complements structural resources by integrating experimentally characterized epitopes with antibody, T-cell and MHC-related information [6].

These databases provide standardized structural, sequence and functional datasets that enable computational modelling, benchmarking, machine learning and large-scale antibody engineering beyond the capabilities of individual experimental studies [1,2,3,4,5,6,7,44,48]. The principal public databases supporting computational antibody engineering, together with their content and major applications, are summarized in Table 1.

### 4.3. Structural Principles Governing Antibody Recognition

#### 4.3.1. Architecture of the Antibody–Antigen-Binding Site

The antibody–antigen-binding site is formed by six complementarity-determining regions (CDRs), comprising three loops from the light chain (L1–L3) and three from the heavy chain (H1–H3) [8,49,50]. These hypervariable loops are supported by a conserved framework, while their sequence, length and spatial arrangement determine antigen specificity by creating a complementary binding surface [8,50,51,52].

Despite extensive sequence diversity, five CDRs (L1–L3, H1 and H2) generally adopt a limited number of recurrent canonical conformations determined primarily by loop length and key structural residues [8,49,50,51,53]. In contrast, CDR-H3 displays substantially greater structural variability and does not conform to the canonical classification [50,54]. Although canonical structures define the backbone geometry, sequence variation at non-conserved positions modifies the physicochemical properties of the antigen-binding surface and contributes to differences in affinity and specificity [8,49,50].

The combination of a conserved structural framework with variable loop conformations enables antibodies to maintain structural stability while recognizing a remarkably broad range of antigens [8,49,50,51].

#### 4.3.2. Canonical Structures of Complementarity-Determining Regions

The concept of canonical structures established that most non-H3 CDRs adopt a limited number of recurrent conformations that can be predicted from loop length and key structural residues [49,51,53]. As larger structural datasets became available, the number of recognized canonical classes increased, confirming the overall validity of the canonical model while revealing additional conformational diversity [51,53].

Expanded structural analyses showed that some CDRs consistently adopt a single conformation, whereas others exhibit multiple sequence-dependent conformations or remain difficult to predict because of structural heterogeneity and interactions with neighboring regions [53]. These findings refined antibody structure prediction by improving classification of CDR conformations and distinguishing predictable from structurally variable loops [53].

Canonical classification is closely linked to antibody numbering systems and CDR definitions [8,49,50,51,53]. Differences among Kabat, Chothia and later structural classification schemes reflect alternative priorities based on sequence variability, structural boundaries or conserved loop geometry [8,49,51,53]. These standardized frameworks provide the structural basis for antibody modelling, comparative analysis and rational antibody engineering [51,53].

#### 4.3.3. Structural Diversity and Biological Importance of CDR-H3

CDR-H3 is the most diverse antibody loop in terms of sequence, length and three-dimensional structure, making it the principal determinant of antigen recognition [50,51,52,54,55]. Unlike the remaining CDRs, its conformation cannot generally be assigned to canonical structural classes because it is influenced by V(D)J recombination, junctional diversity, loop length and interactions with the surrounding framework and neighboring CDRs [50,51,54].

Structurally, CDR-H3 consists of a relatively conserved torso connected to a highly variable head region that adopts diverse conformations [50,53]. Most H3 loops contain a characteristic C-terminal kink that contributes substantially to structural diversity by allowing loops of identical length to adopt different conformations [50,53,54]. Long H3 loops exhibit particularly high conformational variability and frequently assume nonstraight structures stabilized by hydrogen-bonding networks formed before antigen binding [54,55].

The central location of CDR-H3 within the antigen-binding site enables extensive interactions with antigens and neighboring CDRs, making it the dominant contributor to antibody specificity and binding affinity [50,54,55]. Accurate prediction of H3 structure remains one of the greatest challenges in computational antibody modelling and a key objective in rational antibody engineering [54,55].

#### 4.3.4. Antibody Numbering Systems and CDR Definitions

Accurate annotation of antibody variable domains depends on standardized numbering schemes and CDR definitions that enable consistent comparison of antibody sequences and structures [8,10,51]. The Kabat system defines CDRs according to sequence variability, whereas the Chothia scheme incorporates structural information by aligning residue positions with experimentally determined loop conformations [8,10,51].

Subsequent numbering systems further improved standardization. The Martin scheme refined Chothia numbering by resolving inconsistencies associated with insertions and deletions, while the IMGT system introduced a continuous numbering framework based on germline V-gene alignments that facilitates comparisons across different antibody families [10]. These complementary systems provide consistent residue annotation despite differences in antibody sequence length and composition [10].

Because CDR boundaries differ among numbering schemes, the same antibody may contain loops of different lengths depending on the selected annotation method [8,10,51]. Although these differences can influence structural interpretation and computational modelling, standardized numbering systems remain essential for antibody database annotation, structural prediction, comparative analyses and rational antibody engineering [8,10,51].

### 4.4. Physics-Based Computational Antibody Design

#### 4.4.1. Early Computational Approaches to Antibody Structure Prediction

The first computational methods for antibody modelling were based on the observation that antibody variable domains share a conserved structural framework despite substantial sequence diversity [12,13,56]. Early modelling strategies therefore relied on comparative modelling, in which experimentally determined antibody structures served as templates for predicting the three-dimensional structure of related antibodies [12,56].

The accuracy of these approaches depended primarily on the availability of homologous template structures and reliable sequence alignments [12,56]. Conserved framework regions were generally modelled with high precision, whereas prediction of CDR loops, particularly CDR-H3, remained considerably more difficult because of their structural variability [12,13,56]. Antibody modelling workflows evolved into multistep procedures combining template selection, framework construction, loop modelling and structural refinement [12,56].

These early computational approaches demonstrated that experimentally determined antibody structures could be used to predict previously unknown antibody conformations with sufficient accuracy to support structural interpretation and rational engineering [12,13,56]. Although limited by template availability and loop prediction accuracy, comparative modelling established the methodological foundation for subsequent structure-based antibody design and later artificial intelligence-driven prediction methods [12,13,56].

#### 4.4.2. Rational Protein Engineering and Structure-Based Antibody Design

Structure-based antibody engineering emerged from the broader concept of rational protein engineering, in which modifications are guided by structural knowledge rather than random mutagenesis [12,56]. High-resolution three-dimensional structures enabled amino acid substitutions to be designed according to their predicted effects on antibody stability, antigen recognition and intermolecular interactions [12].

Compared with experimental screening alone, structure-based design provided substantially greater control over antibody optimization by allowing multiple structural and energetic constraints to be considered simultaneously [12]. Computational analysis of antibody–antigen interfaces facilitated identification of residues contributing to binding affinity and specificity, enabling targeted modifications that reduced the experimental search space while increasing the probability of obtaining improved variants [12,56].

The availability of expanding structural databases and improved computational algorithms further strengthened rational design by enabling systematic evaluation of sequence–structure relationships before experimental validation [12,13,56]. These developments established structure-guided engineering as an essential component of modern antibody optimization and provided the conceptual framework for subsequent physics-based and artificial intelligence-driven design strategies [12,13,56].

#### 4.4.3. Rosetta and Physics-Based Computational Antibody Engineering

Continued improvements in physics-based modelling methods significantly expanded the capabilities of computational antibody engineering by enabling quantitative evaluation of protein stability, conformational changes and antibody–antigen interactions [13,56]. Among these approaches, the Rosetta framework became one of the most widely used platforms for predicting and optimizing antibody structures through energy-based modelling [13,56].

Rosetta employs an all-atom energy function that combines van der Waals interactions, hydrogen bonding, electrostatics and solvation effects to evaluate protein conformations [13]. Continuous refinement of the scoring function, including improved orientation-dependent solvation models and optimized hydrogen-bonding terms, substantially increased the accuracy of structural prediction and design calculations [13].

Within antibody engineering, Rosetta enabled de novo loop modelling, side-chain optimization, antibody–antigen docking and computational affinity maturation [13,56]. These capabilities allowed candidate mutations to be evaluated before experimental validation, reducing the number of required laboratory experiments while improving the efficiency of rational antibody optimization [56]. Physics-based modelling became a key bridge between traditional structure-guided engineering and the emerging generation of artificial intelligence-based design methods [13,56].

Although Rosetta is commonly described as a physics-based computational framework, its scoring function is not derived exclusively from first-principles physical calculations. Rather, the Rosetta energy function combines physically motivated interaction terms, including van der Waals interactions, electrostatics, hydrogen bonding, and solvation, with empirical and knowledge-based statistical terms [13]. Accordingly, the term “physics-based” in this review refers to the incorporation of physically motivated energetic representations rather than to a strictly first-principles physical model [13].

#### 4.4.4. Computational Optimization of Antibody Affinity and Stability

As computational methods matured, antibody engineering progressed from structural prediction toward rational optimization of antibody function [12,56]. Structural models enabled the identification of residues contributing to antigen recognition, intermolecular interactions and protein stability, allowing mutations to be prioritized according to their predicted energetic effects before experimental validation [12,56].

Energy-based calculations and structural refinement facilitated the evaluation of amino acid substitutions that could improve binding affinity while maintaining conformational stability [13,56]. Rather than relying on exhaustive experimental screening, computational design reduced the number of candidate variants by focusing on mutations with the highest predicted probability of success, thereby increasing the efficiency of antibody optimization [12,56].

Although these approaches remained dependent on accurate structural models and computational scoring functions, they demonstrated that antibody properties could be systematically optimized through iterative cycles of modelling and experimental validation [13,56]. This transition from structure prediction to rational computational design established the methodological foundation for the deep learning and generative artificial intelligence approaches described in subsequent sections [12,13,56].

### 4.5. Deep Learning Revolution in Antibody Structure Prediction

#### 4.5.1. Deep Learning for Antibody Structure Prediction

The application of deep learning marked a major transition in computational antibody engineering by replacing manually designed structural rules with data-driven prediction models trained on large structural datasets [57,58,59]. Unlike traditional template-based methods, deep learning algorithms learned complex relationships between antibody sequences and three-dimensional structures directly from experimental data, substantially improving prediction accuracy while reducing computational time [57,58].

Several antibody-specific architectures were subsequently developed to address the limitations of conventional modelling, particularly for highly variable CDR loops [58,59,60]. DeepAb combined interpretable deep neural networks with structural modelling to improve prediction of inter-residue geometries and CDR conformations, whereas ABlooper employed equivariant graph neural networks for rapid and accurate prediction of antibody loop structures, including CDR-H3 [58,59]. These approaches demonstrated that deep learning could outperform conventional modelling strategies for structurally challenging antibody regions [58,59].

The integration of deep learning into antibody modelling established the foundation for modern computational antibody engineering by providing highly accurate structural predictions suitable for downstream applications such as affinity optimization, developability assessment and generative antibody design [57,58,59,60].

#### 4.5.2. AlphaFold and the Transformation of Protein Structure Prediction

The release of AlphaFold represented a major breakthrough in computational structural biology by demonstrating that deep learning could predict protein structures with near-experimental accuracy for many proteins [60,61,62]. By learning sequence–structure relationships from large structural datasets, AlphaFold substantially outperformed previous template-based and physics-driven modelling approaches while greatly reducing prediction time [60,62].

Although AlphaFold was not developed specifically for antibodies, its architecture and prediction strategy profoundly influenced computational antibody engineering [60,61]. The high accuracy achieved for conserved framework regions confirmed the potential of end-to-end deep learning for protein structure prediction, while persistent difficulties in modelling highly flexible CDR-H3 loops highlighted the unique challenges posed by antibodies [60,61].

The success of AlphaFold accelerated the development of antibody-specific deep learning models that incorporated structural, evolutionary and antibody-focused datasets to improve prediction of variable regions [58,59,60,61,62]. AlphaFold not only transformed general protein structure prediction but also established the methodological framework for the next generation of artificial intelligence tools dedicated to antibody modelling and design [58,59,60,61,62].

#### 4.5.3. Antibody-Specific Deep Learning Models

The limitations of general protein prediction models in highly variable antibody regions stimulated ongoing developments in antibody-specific deep learning architectures trained on immunoglobulin structural datasets [58,59,60,63]. These models incorporated antibody-specific structural constraints to improve prediction of CDR conformations, particularly the highly diverse CDR-H3 loop, which remained the principal source of modelling error in conventional approaches [58,59,60].

DeepAb introduced an interpretable deep learning framework that predicts inter-residue geometries prior to three-dimensional structure reconstruction, improving both structural accuracy and model interpretability [58]. ABlooper further increased prediction speed by employing equivariant graph neural networks capable of directly modelling CDR loop conformations while preserving rotational and translational invariance [59]. Additional antibody-oriented architectures progressively incorporated graph representations and geometric learning strategies to enhance prediction of local structural features and antibody flexibility [59,63].

These antibody-specific models demonstrated that incorporating domain-specific structural knowledge substantially improves prediction accuracy compared with general-purpose protein modelling approaches [58,59,60,63]. Their success established deep learning as the dominant strategy for computational antibody structure prediction and created the foundation for subsequent generative models capable of designing entirely new antibody sequences and structures [58,59,60,63].

#### 4.5.4. Computational Prediction of Antibody Developability

As antibody therapeutics progressed toward clinical application, computational modelling expanded beyond structural prediction to include assessment of antibody developability [16,17,18,19]. In addition to high antigen affinity, therapeutic antibodies must exhibit favorable physicochemical properties, including adequate stability, solubility, manufacturability and low aggregation propensity [16,17].

Computational developability assessment integrates structural, sequence-based and biophysical descriptors to identify liabilities early in the design process [16,17,18,19]. Predictive models evaluate features associated with aggregation, hydrophobic surface patches, charge distribution, thermal stability and other molecular characteristics that influence production, formulation and clinical performance [16,17]. Early identification of unfavorable properties enables candidate molecules to be optimized before experimental development, thereby reducing costs and improving the probability of successful translation [16,17,18,19].

The incorporation of developability prediction into computational antibody engineering shifted the focus from optimizing binding affinity alone toward simultaneous optimization of multiple therapeutic properties [16,17,18,19]. This multidimensional strategy represents an important step toward integrated artificial intelligence frameworks capable of balancing affinity, stability, manufacturability and safety during antibody design [16,17,18,19].

### 4.6. Protein Language Models in Antibody Engineering

#### 4.6.1. Foundations of Protein Language Models

Protein language models (PLMs) represent a new generation of artificial intelligence methods that learn sequence representations from large collections of protein sequences using self-supervised learning [57,58,64]. Inspired by natural language processing, these models treat amino acid sequences as biological “sentences,” enabling them to capture statistical relationships that reflect structural, functional and evolutionary properties without requiring explicit structural annotations [57,64].

Unlike conventional feature-engineering approaches, PLMs generate contextual embeddings in which each amino acid is represented according to its sequence environment [57,58,64]. These embeddings encode biologically relevant information associated with protein folding, residue interactions and functional constraints, making them suitable for a broad range of downstream tasks, including structure prediction, mutation-effect prediction and antibody engineering [57,64].

The availability of increasingly large protein-sequence databases substantially improved model performance by enabling language models to learn general principles of protein organization directly from evolutionary sequence diversity [57,58,64]. Protein language models established a versatile computational framework that complements structural modelling while providing the foundation for antibody-specific language models described in the following sections [57,58,64].

#### 4.6.2. General Protein Language Models: ESM and ProtBERT

Early protein language models demonstrated that representations learned from unlabeled protein sequences could capture biologically meaningful information applicable to a wide range of computational tasks [57,64,65]. Models such as ESM and ProtBERT were trained on millions of protein sequences using transformer architectures, enabling them to learn sequence patterns associated with protein structure, function and evolution without requiring experimentally determined structural labels [57,64,65].

These general-purpose models generate contextual embeddings that can be transferred to downstream applications through transfer learning [57,64]. The learned representations have been successfully applied to protein structure prediction, residue-property prediction, functional annotation and mutation-effect analysis, often outperforming conventional sequence-based descriptors [57,64,65].

Although developed for the entire protein universe rather than antibodies specifically, general protein language models demonstrated that evolutionary information embedded in large sequence databases can be effectively exploited through self-supervised learning [57,64,65]. Their success provided the conceptual and methodological foundation for antibody-specific language models designed to capture the unique structural and functional characteristics of immunoglobulin repertoires [57,58,64,65].

#### 4.6.3. Antibody-Specific Language Models: AntiBERTa and AbLang

The success of general protein language models stimulated the development of antibody-specific architectures trained exclusively on immunoglobulin repertoires [20,58,64,65]. By learning from antibody sequence diversity rather than the complete protein universe, these models captured sequence patterns associated with antibody maturation, CDR organization and functional specialization more effectively than general-purpose language models [20,58,64].

Among the most widely used antibody-specific models, AntiBERTa applies transformer-based self-supervised learning to large B-cell receptor repertoires, generating contextual embeddings that encode biologically meaningful features of antibody sequences [20]. Similarly, AbLang was designed to learn antibody-specific sequence representations that improve downstream applications, including sequence completion, mutation analysis and antibody characterization [23].

Compared with general protein language models, antibody-specific architectures consistently provide richer representations of immunoglobulin sequence space by incorporating the unique evolutionary and structural characteristics of antibody repertoires [20,23,58,64]. These models have become valuable tools for antibody classification, engineering and downstream artificial intelligence applications that require accurate sequence-level representations [20,23,58,64].

#### 4.6.4. Embeddings and Transfer Learning in Antibody Engineering

A key advantage of protein language models is their ability to generate embeddings, numerical representations that encode structural, functional and evolutionary information learned directly from protein sequences [57,64,65]. Unlike conventional handcrafted descriptors, embeddings capture complex sequence relationships in a high-dimensional space, providing informative features for diverse downstream prediction tasks [57,64].

These representations are commonly applied through transfer learning, in which a pretrained language model serves as the foundation for a task-specific model requiring only limited additional training [57,64]. This strategy enables knowledge acquired from millions of protein sequences to be transferred efficiently to antibody-related applications, including structure prediction, mutation-effect prediction, functional annotation and developability assessment [20,23,57,64].

By reducing the dependence on large labeled datasets, transfer learning has substantially expanded the applicability of artificial intelligence in antibody engineering [57,64]. Embeddings and transfer learning bridge large-scale self-supervised learning with specialized antibody prediction tasks, establishing protein language models as a central component of modern computational antibody engineering [20,23,57,58,64,65]. Representative protein language models used in antibody engineering, together with their architectures, applications, and major advantages, are summarized in Table 2.

### 4.7. Generative Artificial Intelligence for De Novo Antibody Design

#### 4.7.1. Generative Artificial Intelligence in Antibody Engineering

Generative artificial intelligence represents a fundamental shift in computational antibody engineering by moving beyond prediction and optimization toward the de novo generation of antibody sequences and structures [67,68,69]. Unlike conventional machine-learning approaches, which primarily evaluate existing molecules, generative models learn the underlying probability distribution of antibody sequence and structural space, enabling the creation of novel antibody candidates with predefined characteristics [67,68].

Early generative approaches were based on variational autoencoders (VAEs) and generative adversarial networks (GANs), which demonstrated that biologically plausible antibody sequences could be generated while preserving key structural and functional features [68,69]. These methods enabled exploration of previously inaccessible regions of sequence space and illustrated the potential of artificial intelligence to accelerate antibody discovery beyond experimental repertoire screening [68].

The introduction of generative models expanded computational antibody engineering from rational optimization to molecular design [67,68,69]. By integrating structural information, evolutionary constraints and functional objectives into a unified framework, generative artificial intelligence established the foundation for the diffusion-based approaches that currently represent the state of the art in antibody design [67,68,69].

#### 4.7.2. Variational Autoencoders and Generative Adversarial Networks

Variational autoencoders (VAEs) and generative adversarial networks (GANs) were among the first deep generative architectures applied to antibody engineering [68,69]. These models demonstrated that antibody sequence space could be represented as a continuous latent distribution from which novel antibody candidates could be generated while preserving biologically meaningful sequence patterns [68].

VAEs encode antibody sequences into a latent space that captures essential structural and functional characteristics before reconstructing or generating new sequence variants [68,69]. This continuous representation enables interpolation between antibodies and systematic exploration of sequence diversity, facilitating the design of molecules with desired functional properties [68]. In contrast, GANs employ adversarial training between a generator and a discriminator, producing increasingly realistic antibody sequences through iterative optimization [69].

Although these early generative models demonstrated the feasibility of de novo antibody design, they remained limited by challenges associated with structural consistency, training stability and precise control over generated molecules [68,69]. Nevertheless, VAEs and GANs established the conceptual basis for subsequent diffusion-based generative models, which substantially improved structural accuracy and controllability in computational antibody design [67,68,69].

#### 4.7.3. Diffusion-Based Generative Models for Antibody Engineering

The introduction of diffusion models represented a major breakthrough in generative antibody design by enabling the simultaneous generation of antibody sequences and three-dimensional structures while explicitly conditioning the design process on antigen geometry [67,68,69,70,71]. Unlike earlier generative architectures, diffusion models iteratively refine noisy molecular representations into structurally realistic antibodies, providing greater stability, controllability and structural accuracy [67,68,69].

Several antibody-specific diffusion frameworks have subsequently been developed. DiffAb introduced conditional generation of antibody structures guided by antigen information, enabling targeted design of complementary binding interfaces [67]. AbDiffuser further incorporated structural priors to improve conformational realism during antibody generation, whereas AbX integrated evolutionary and structural information to enhance prediction quality and sequence diversity [70,71]. More recently, RFdiffusion-antibody extended diffusion-based design by combining geometric deep learning with structure-aware generation, allowing antibodies to be designed directly around predefined antigen epitopes [69].

Diffusion-based models substantially expanded the capabilities of computational antibody engineering by integrating structural prediction, sequence generation and antigen-specific conditioning within a unified generative framework [67,68,69,70,71]. These advances established diffusion models as the current state-of-the-art approach for de novo antibody design and a major step toward fully automated therapeutic antibody engineering [67,69,70,71].

#### 4.7.4. Reinforcement Learning and Multi-Objective Antibody Optimization

The latest generation of artificial intelligence methods has extended generative antibody design by incorporating reinforcement learning and multi-objective optimization strategies [69,70,71]. Rather than generating antibodies solely on the basis of structural plausibility, these approaches iteratively optimize candidate molecules according to predefined objectives, including binding affinity, structural stability, developability and antigen specificity [69,70].

Reinforcement learning enables generative models to refine antibody sequences through feedback obtained from predictive scoring functions, progressively favoring variants with improved predicted properties [69]. In parallel, multi-objective optimization balances competing design constraints, allowing several therapeutic characteristics to be optimized simultaneously instead of maximizing a single parameter such as binding affinity [70,71].

The integration of reinforcement learning with diffusion-based generative models represents an important step toward autonomous antibody engineering [69,70,71]. By combining de novo sequence generation with iterative optimization under multiple structural and functional constraints, these approaches move computational antibody design closer to fully automated discovery of therapeutic antibodies with experimentally relevant properties [69,70,71]. The principal generative artificial intelligence approaches currently used for antibody engineering are compared in Table 3.

### 4.8. AI-Guided Antibody Optimization

#### 4.8.1. Artificial Intelligence for Antibody Humanization

One of the earliest clinical applications of artificial intelligence in antibody engineering has been antibody humanization, where computational methods aim to reduce immunogenicity while preserving antigen-binding properties [80,81,82]. Traditional humanization relied primarily on framework grafting followed by extensive experimental optimization, whereas artificial intelligence enables systematic identification of human framework sequences that maintain structural integrity and binding-site geometry [80,81].

Machine-learning models predict the effects of framework substitutions on antibody stability, structural conformation and antigen recognition, allowing unfavorable mutations to be excluded before laboratory validation [80,81,82]. By integrating sequence, structural and evolutionary information, these approaches reduce the number of experimental design cycles while improving the probability of retaining functional activity after humanization [80,81].

The incorporation of artificial intelligence into antibody humanization has therefore transformed a largely empirical process into a rational, data-driven workflow [80,81,82]. Beyond reducing immunogenicity, these methods contribute to the evolution of therapeutic antibodies with improved developability and a greater likelihood of successful clinical translation [80,81,82].

#### 4.8.2. Artificial Intelligence for Affinity Maturation: AI-Guided Antibody Optimization

Artificial intelligence has substantially improved computational affinity maturation by enabling prediction of mutations that enhance antibody–antigen interactions while minimizing experimental screening [83,84,85,86]. Unlike conventional affinity maturation, which relies on iterative mutagenesis and selection, machine-learning approaches prioritize sequence variants according to their predicted effects on binding affinity and structural stability [83,84].

Deep learning and structure-informed predictive models evaluate the energetic and structural consequences of amino acid substitutions within antibody variable domains, identifying mutations with the highest probability of improving antigen recognition [83,84,85]. By integrating sequence, structural and experimental data, these methods efficiently explore the extensive mutational landscape while substantially reducing the number of variants requiring laboratory validation [83,84,85,86].

The application of artificial intelligence has therefore transformed affinity maturation into a predictive optimization process in which computational models guide experimental design [83,84,85,86]. This integration accelerates antibody optimization while improving the efficiency of therapeutic antibody development through more targeted exploration of sequence space [83,84,85,86].

### 4.9. Beyond Affinity Optimization

#### 4.9.1. Artificial Intelligence for Antibody Engineering Beyond Affinity Optimization

The rapid development of artificial intelligence has expanded computational antibody engineering beyond affinity optimization toward comprehensive modulation of therapeutic antibody properties [58,87,88,89]. Modern artificial intelligence frameworks simultaneously consider multiple molecular characteristics, including specificity, stability, solubility, pharmacokinetics and immunogenicity, enabling antibody optimization to be performed within an integrated computational workflow rather than through sequential experimental refinement [87,88,89].

This transition reflects a broader change in antibody engineering, where therapeutic performance is increasingly viewed as the result of multiple interacting molecular properties rather than binding affinity alone [87,88]. By combining structural modelling, sequence analysis and machine-learning predictions, artificial intelligence enables early identification of design strategies that balance diverse therapeutic requirements while reducing experimental screening efforts [58,88,89].

Artificial intelligence has evolved from a tool for improving antibody–antigen interactions into a comprehensive platform supporting the optimization of antibody function, developability and clinical potential [58,87,88,89]. This broader perspective has established the foundation for specialized artificial intelligence applications targeting individual therapeutic properties discussed in the following sections [88,89]. The historical evolution of computational antibody engineering, together with the dominant computational paradigms, technological advances, achievements, and remaining limitations, is summarized in Table 4.

#### 4.9.2. Artificial Intelligence for Engineering Antibody Specificity and Selectivity

Artificial intelligence has increasingly been applied to optimize antibody specificity by improving discrimination between target and off-target antigens [58,89,90,91]. Computational models integrate sequence, structural and binding-interface information to predict mutations that enhance target recognition while minimizing undesired cross-reactivity, thereby improving both therapeutic efficacy and safety [58,89].

Machine-learning approaches analyze antibody–antigen interactions at multiple levels, including residue contacts, structural complementarity and physicochemical features, enabling identification of determinants responsible for antigen selectivity [89,90]. These predictions support rational modification of antibody variable regions while reducing the need for extensive experimental screening of large mutational libraries [58,89,91].

The ability to computationally optimize specificity represents an important extension of artificial intelligence beyond affinity prediction alone [58,89,90,91]. By simultaneously considering target recognition and off-target interactions, artificial intelligence contributes to the advancement of therapeutic antibodies with improved functional precision and reduced risk of unintended biological activity [58,89,90,91].

#### 4.9.3. Artificial Intelligence for Optimizing Antibody Stability and Physicochemical Properties

Artificial intelligence has become an important tool for optimizing antibody stability and other physicochemical properties that influence therapeutic performance beyond antigen recognition [91,92,93,94]. Machine-learning models predict the effects of sequence modifications on structural stability, aggregation propensity, solubility and conformational integrity, enabling unfavorable variants to be identified before experimental validation [91,92].

Computational optimization integrates sequence- and structure-based features to evaluate how individual mutations influence multiple physicochemical characteristics simultaneously [91,93]. These predictive frameworks facilitate rational design by prioritizing mutations that improve molecular robustness while preserving antibody function, thereby reducing the experimental effort required for candidate optimization [92,93,94].

The addition of artificial intelligence into stability engineering extends computational antibody design beyond binding optimization toward the refinement of molecules with favorable biophysical properties suitable for therapeutic application [91,92,93,94]. As a result, stability prediction has become an integral component of modern antibody engineering workflows, complementing affinity, specificity and developability optimization [91,92,93,94].

#### 4.9.4. Artificial Intelligence for Optimizing Pharmacokinetic Properties and Immunogenicity

Artificial intelligence has also been applied to optimize pharmacokinetic properties and reduce the immunogenicity of therapeutic antibodies, extending computational engineering beyond molecular recognition and structural stability [95,96,97,98]. Machine-learning models analyze sequence, structural and physicochemical features associated with antibody persistence, clearance and immune recognition, enabling early prediction of potential liabilities during antibody development [95,96].

Computational approaches identify sequence modifications that preserve antibody function while minimizing the probability of undesirable immune responses or unfavorable pharmacokinetic behavior [95,97]. By integrating multiple molecular descriptors into predictive frameworks, artificial intelligence supports rational optimization of therapeutic antibodies before experimental testing, reducing development time and increasing the likelihood of successful clinical translation [95,96,97,98].

The incorporation of pharmacokinetic and immunogenicity prediction into artificial intelligence-driven antibody engineering reflects the transition from optimizing individual molecular properties toward comprehensive therapeutic design [95,96,97,98]. Together with advances in affinity, specificity, stability and developability prediction, these approaches move computational antibody engineering closer to fully integrated end-to-end design of clinically relevant antibody therapeutics [95,96,97,98].

#### 4.9.5. Inverse Folding and Sequence–Structure Co-Design in Antibody Engineering

Inverse folding represents a structure-conditioned approach to protein design in which an amino acid sequence is generated for a predefined backbone structure [99,100]. In antibody engineering, this strategy is particularly relevant because preservation of the antibody fold is essential for maintaining antigen-binding properties, although its applicability may be constrained by the limited availability of accurate antibody–antigen structures [100,101]. ProteinMPNN is a message-passing neural network developed for protein sequence design from backbone structural information and has demonstrated substantially higher native sequence recovery than Rosetta while requiring considerably less computational time [99]. Its general applicability and computational efficiency have contributed to its use as an important component of contemporary protein-design workflows [99].

ProteinMPNN also provided a foundation for antibody-specific inverse-folding approaches. AbMPNN was developed by fine-tuning ProteinMPNN on antibody-focused datasets and is designed to predict and generate antibody sequences, including variable CDRs [100,101]. More broadly, inverse-folding models can enrich higher-affinity antibodies from candidate pools and can therefore support filtering of sequences obtained from display, immunization, or other discovery workflows [100]. Nevertheless, their performance in antibody engineering remains constrained by limited incorporation of antigen information, with structural constraints often dominating over functional signals related to the antibody–antigen interface [100]. General models such as ProteinMPNN may therefore generate structurally plausible sequences without fully capturing the chemical characteristics required for antigen-specific binding [100].

Sequence–structure co-design represents a more integrated alternative to fixed-backbone inverse folding by allowing structural and sequence properties to be optimized simultaneously in the context of a desired target [101]. BoltzGen exemplifies this class of models as a unified all-atom diffusion framework that combines structure prediction and binder design and can simultaneously generate amino-acid identities and all-atom structures [101,102]. The model can additionally incorporate target-related constraints, including binding-site information, and can generate nanobody CDRs while maintaining a predefined framework [102]. In this sense, BoltzGen reflects the broader transition from fixed-backbone sequence optimization toward end-to-end generative systems integrating target conditioning, structure generation, and sequence design [101].

BoltzGen has also undergone experimental validation. Nanomolar-affinity nanobody binders were obtained for six of nine novel targets lacking closely related bound complexes in the PDB, providing evidence that the approach can generalize beyond familiar structural templates [101,102]. However, co-design does not eliminate the need for subsequent filtering and experimental evaluation, because high predicted affinity alone does not ensure adequate selectivity, developability, or therapeutic performance [102]. Moreover, reported limitations such as reduced generation diversity and sequence memorization illustrate that unified generative models remain susceptible to biases originating from their training data [102]. Thus, inverse folding and sequence–structure co-design should be regarded as complementary components of computational antibody engineering: the former efficiently identifies sequences compatible with predefined structures, whereas the latter extends the design space by jointly considering sequence, structure, and target-dependent interactions [100,101,102].

The representative AI models discussed throughout this review are summarized in Table 5, together with their AI approaches, primary applications, distinctive features, and representative references.

### 4.10. Quantitative Performance and Experimental Validation of AI-Based Antibody Engineering Methods

Quantitative evaluation of AI-based antibody engineering methods varies considerably across individual computational tasks, with the most standardized comparisons currently available for antibody structure prediction. On the 49-antibody Rosetta Antibody Benchmark, mean CDR-H3 RMSD values were 2.87 Å for AlphaFold2, 2.77 Å for ABodyBuilder, 2.44 Å for DeepAb, and 2.49 Å for ABlooper [18]. For ABlooper, restricting the analysis to the 75% most confident predictions further reduced the mean CDR-H3 RMSD from 2.49 to 2.05 Å, illustrating the potential value of model-specific confidence estimation [18]. Independent evaluation of IgFold used a temporally separated benchmark to minimize overlap with training structures and showed high framework accuracy across antibody-specific models, with mean heavy- and light-chain framework RMSDs of 0.43–0.53 Å and 0.41–0.51 Å, respectively [22]. Nevertheless, performance remains less consistent for conformationally variable regions; for nanobody CDR3 prediction, AlphaFold and IgFold achieved mean RMSDs of 4.00 Å and 4.25 Å, respectively, whereas DeepAb reached 8.52 Å [22]. These results also illustrate the dependence of model performance on the composition of the training data and the structural space represented during training [22].

Quantitative assessment of antibody developability is substantially more heterogeneous because individual models target different physicochemical and biological endpoints. The Therapeutic Antibody Developability Analysis (TA-DA) model combined five selected sequence- and structure-based descriptors and achieved an AUC of 0.80 for distinguishing clinical antibodies from repertoire antibodies in a held-out test set [103]. However, correlations with experimental polyspecificity assays were relatively modest, with rank-order correlations generally between 0.2 and 0.3, emphasizing that computational developability scores should not be interpreted as substitutes for experimental characterization [103]. This limitation is reinforced by the dependence of four of the five TA-DA descriptors on predicted antibody structures, directly linking downstream developability assessment to the accuracy of structural modelling [103].

A broader quantitative perspective is provided by the FLAb2 benchmark, which comprises experimental developability data for more than four million antibodies collected across 32 studies and evaluates 30 AI and biophysical models across thermostability, expression, aggregation, binding affinity, pharmacokinetics, polyreactivity, and immunogenicity [104]. Protein AI models produced statistically significant correlations for only a minority of developability datasets overall, and no single model demonstrated uniformly strong performance across all properties [104]. In zero-shot evaluation, expression was comparatively more predictable, with an average Spearman correlation of ρ = 0.22, whereas aggregation yielded an average ρ = −0.07 [104]. Individual best-performing models also differed by endpoint: Chai-1 reached ρ = 0.45 for thermostability, ESM2 150M reached ρ = 0.44 for expression, BioPython charge at pH 7.4 reached ρ = 0.59 for pharmacokinetics, and ProGen2 Medium reached ρ = 0.34 for immunogenicity [104]. Binding prediction likewise remained challenging, with AI models showing statistically significant correlations in only approximately 20% of the evaluated binding datasets [104]. These findings indicate that quantitative performance in one property cannot be assumed to generalize to other aspects of antibody developability [104].

Experimental validation remains particularly important for generative antibody-design methods because favorable in silico scores do not necessarily establish functional antibody production or antigen binding. AbDiffuser provides one example in which computational generation was followed by direct laboratory validation: all 16 submitted designs were successfully expressed as antibodies, 37.5% bound HER2, and the experimentally confirmed binders had an average pKD of 8.7 [68]. Additional computational filtering increased the binding rate from 22.2% among unfiltered designs to 57.1% among filtered designs, with corresponding average pKD values of 8.53 and 8.78 [68]. The tightest experimentally measured AbDiffuser binder achieved affinity comparable to or slightly exceeding that of trastuzumab, demonstrating that computational filtering can substantially enrich experimentally functional candidates while still requiring direct wet-lab confirmation [68].

An even larger experimental assessment was reported for zero-shot generative antibody design against HER2. The generated HCDR3 and HCDR123 sequences achieved experimental binding rates of 10.6% and 1.8%, respectively, corresponding to four- and eleven-fold improvements over randomly sampled OAS baselines [105]. Antigen specificity was also experimentally supported by a significant reduction in binding rates when incorrect antigens, including rat HER2, HER3, or VEGF, were supplied to the model [105]. Among 421 AI-designed HER2 binders subsequently confirmed using surface plasmon resonance, 71 exhibited affinities below 10 nM, three bound more tightly than trastuzumab, and one achieved sub-nanomolar affinity [105]. Experimental validation on additional targets identified a VEGF-A-binding design with a KD of 48.2 nM and an Omicron spike RBD-binding design with a KD of 179.7 nM; the latter showed no detectable binding to other tested spike variants, further supporting the potential for target-specific generative design [105].

Overall, the available evidence shows a clear distinction between computational benchmarking and experimental validation. Structure-prediction methods can increasingly be compared using shared RMSD-based benchmarks, whereas affinity and developability prediction remain more dependent on heterogeneous datasets, endpoints, and experimental assays [18,22,103,104]. For generative antibody design, several recent studies have progressed beyond purely computational evaluation by experimentally confirming antibody expression, antigen binding, affinity, and, in selected cases, specificity [68,105]. However, the experimental evidence summarized here primarily concerns in vitro functional validation rather than demonstration of in vivo efficacy or clinical therapeutic activity, indicating that substantial validation remains necessary before AI-generated antibodies can be considered clinically established therapeutic candidates [68,105].

## 5. Discussion

### 5.1. Evolution from Experimental to Computational Antibody Engineering

The evolution of computational antibody engineering reflects a progressive transition from experimentally driven optimization toward integrated artificial intelligence-based design. Early advances were driven by phage display, combinatorial antibody libraries and in vitro affinity maturation, which enabled efficient selection and optimization of antigen-specific antibodies [24,25,27,29]. The subsequent introduction of high-throughput sequencing, standardized antibody numbering systems and publicly available structural databases provided the large-scale datasets necessary for computational modelling and data-driven antibody engineering [1,5,37,44].

The transition from empirical antibody discovery to rational computational design was initially enabled by structure-based modelling approaches. Comparative modelling, molecular docking and physics-based methods, particularly Rosetta, demonstrated that antibody structures and antibody–antigen interactions could be predicted with sufficient accuracy to guide targeted sequence optimization before experimental validation [12,13,56]. Nevertheless, these approaches remained limited by template availability, computational cost and the intrinsic structural complexity of highly flexible regions such as CDR-H3 [10,54,56].

### 5.2. Deep Learning and Generative AI in Antibody Engineering

Deep learning fundamentally changed computational antibody modelling by learning sequence–structure relationships directly from experimental datasets rather than relying on manually designed structural rules. Antibody-specific architectures, including DeepAb and ABlooper, together with advances inspired by AlphaFold, substantially improved structural prediction, particularly for antibody variable domains [58,59,60,61,62]. In parallel, protein language models demonstrated that biologically meaningful sequence representations could be learned through self-supervised training on large protein and antibody repertoires, providing transferable embeddings for numerous downstream antibody-engineering applications [20,23,57,64].

The emergence of generative artificial intelligence represents the most recent milestone in computational antibody engineering. Unlike predictive models that evaluate existing antibodies, generative approaches enable the de novo design of antibody sequences and structures while integrating structural constraints, antigen information and functional objectives [67,68,69]. Diffusion-based architectures, including DiffAb, AbDiffuser, AbX and RFdiffusion-antibody, have further expanded these capabilities by combining sequence generation, structural modelling and antigen-conditioned optimization within a unified framework [67,69,70,71].

### 5.3. Multi-Parameter Optimization and Current Limitations

An equally important trend identified throughout this review is the expansion of artificial intelligence beyond affinity optimization alone. Contemporary computational frameworks increasingly address antibody specificity, stability, solubility, developability, pharmacokinetic behaviour and immunogenicity, reflecting the multidimensional nature of therapeutic antibody development [80,89,95,106]. Rather than optimizing individual properties independently, recent artificial intelligence models integrate multiple predictive objectives to identify antibody candidates with balanced therapeutic characteristics before experimental validation [107,108,109,110,111].

Despite these advances, several important limitations remain. Most artificial intelligence models continue to depend heavily on the availability, quality and diversity of experimentally determined sequence and structural datasets, which may introduce biases and restrict model generalizability [5,44,58,64]. Furthermore, accurate prediction of highly flexible CDR-H3 conformations remains considerably more challenging than modelling conserved framework regions, and computational predictions still require rigorous experimental verification before therapeutic application [54,55,58,60].

### 5.4. Future Perspectives in AI-Based Antibody Engineering

Future progress in antibody engineering will likely depend on closer integration of experimental and computational methodologies. The combination of high-throughput repertoire sequencing, structural biology, protein language models, diffusion-based generative architectures and reinforcement learning is expected to enable increasingly automated and efficient antibody-design workflows [57,64,68,69]. Rather than replacing experimental antibody engineering, artificial intelligence is emerging as a complementary technology that prioritizes promising candidates, accelerates optimization and reduces experimental workload while supporting the progress of next-generation therapeutic antibodies [58,67,69,107].

### 5.5. Failure Modes and Limitations of AI-Based Antibody Engineering

Despite the rapid progress of artificial intelligence in antibody engineering, current models remain susceptible to several methodological and biological failure modes that may lead to an overestimation of their practical performance. Data leakage and benchmark contamination represent particularly important concerns, as overlap between training datasets and commonly used structural or sequence benchmarks can artificially inflate reported predictive accuracy. This issue is especially relevant in antibody modelling, where available experimentally determined structures remain relatively limited and are repeatedly incorporated into successive databases and training datasets. Consequently, high performance on retrospective benchmarks does not necessarily indicate equivalent generalizability to previously unseen antibodies.

Training-set bias constitutes a related limitation. Models trained predominantly on naturally occurring antibodies may preferentially reproduce features characteristic of germline or repertoire sequences rather than properties directly associated with therapeutic developability. Such models may therefore perform less reliably when applied to extensively engineered antibodies or sequence–structure combinations that differ substantially from their training distributions. Distribution shift may become even more pronounced when models are applied to uncommon antibody formats, unusual CDR-H3 conformations, novel antigens, or targets that are poorly represented in available datasets. Antigen-conditioned models are additionally vulnerable to epitope bias when training data disproportionately represent particular antigen classes or experimentally accessible epitopes.

Generative approaches introduce further challenges because computationally plausible sequences are not necessarily experimentally functional antibodies. Generated sequences may satisfy learned statistical or structural constraints while exhibiting inadequate expression, stability, specificity, or other properties required for therapeutic development. Computational filtering can reduce this risk but remains dependent on the accuracy and applicability of the predictive models used for candidate selection. Uncertainty estimation is therefore particularly important, as apparently precise predictions may conceal substantial model uncertainty, especially for antibodies located outside the distribution represented during training.

Most importantly, predicted antibody properties should not be considered equivalent to experimentally demonstrated properties. Computational estimates of affinity, stability, developability, or structural quality can prioritize candidates and reduce experimental search space, but experimental confirmation remains necessary to establish actual molecular behaviour. Recent experimental validation of AI-generated antibodies demonstrates that generative approaches can produce functional binders, yet evidence remains predominantly based on in vitro characterization, while comprehensive developability assessment, in vivo efficacy, safety, and clinical validation remain considerably less established. These limitations emphasize that computational performance should be interpreted within the context of dataset composition, benchmark independence, model uncertainty, and the level of experimental evidence supporting individual predictions.

### 5.6. Generative AI as an Integrated Antibody Design Workflow

The most recent generative approaches are increasingly shifting antibody engineering from isolated prediction tasks toward integrated design workflows. Within such a framework, sequence generation and structure generation represent complementary stages rather than independent objectives, because alterations in antibody sequence directly influence structural organization and functional properties. Joint sequence–structure modelling may therefore provide a more coherent representation of the design problem than sequential approaches in which structural and sequence information are optimized independently.

Antigen-conditioned design introduces an additional level of functional control by incorporating information about the intended target during antibody generation. Candidate antibodies can subsequently undergo affinity optimization and computational developability filtering to prioritize sequences predicted to combine target recognition with acceptable physicochemical characteristics. These computational stages should be viewed as candidate-enrichment procedures rather than endpoints of antibody development. Experimental screening remains necessary to determine whether generated candidates are expressed correctly and exhibit the predicted binding, specificity, affinity, and developability characteristics.

The greatest potential of generative AI may therefore lie in iterative model–experiment cycles rather than fully autonomous in silico antibody design. Experimental results can provide feedback for subsequent rounds of computational generation and optimization, progressively restricting the design space toward candidates with experimentally supported properties. Such integration creates a workflow extending from sequence and structure generation through antigen-conditioned design, affinity optimization and developability filtering to experimental screening and subsequent model-guided refinement.

The transition from predictive modelling to generative antibody engineering should not be interpreted as the replacement of experimental discovery by artificial intelligence. Instead, the emerging paradigm is a closed interaction between computational design and experimental validation, in which each stage addresses limitations of the other. The clinical value of generative AI will therefore depend not only on increasingly sophisticated models, but also on the quality and independence of their training and benchmark data, reliable uncertainty estimation, rigorous experimental validation, and demonstration that computationally optimized antibodies retain their predicted properties under biologically and clinically relevant conditions.

## 6. Conclusions

Ongoing progress in computational antibody engineering reflects a progressive transition from experimentally guided optimization toward increasingly autonomous artificial intelligence-driven design. Early structure-based approaches provided the first rational framework for predicting antibody structures and prioritizing mutations, but their performance depended heavily on experimentally determined templates, manually designed scoring functions, and computationally intensive simulations. The rapid expansion of structural databases and high-throughput sequencing subsequently created the large-scale datasets required for modern data-driven modelling.

The emergence of deep learning fundamentally changed antibody engineering by enabling accurate prediction of antibody structures directly from sequence information and improving the modelling of structurally complex regions. Protein language models further expanded these capabilities by learning biologically meaningful sequence representations from millions of antibody and protein sequences, facilitating a wide range of downstream applications, including structure prediction, developability assessment, and functional annotation. More recently, generative artificial intelligence has extended computational antibody engineering beyond prediction toward the design of entirely new antibody sequences and structures conditioned on desired molecular properties.

Despite these advances, several important challenges remain. Limited experimental validation, biases in available datasets, prediction of highly flexible antibody regions, model interpretability, and the translation of computational predictions into clinically successful therapeutics continue to limit the full potential of current AI-based approaches. Addressing these limitations will require closer integration of computational modelling with experimental validation and standardized benchmarking across diverse datasets.

Overall, the field is rapidly evolving toward integrated AI-driven platforms capable of combining structural prediction, sequence optimization, developability assessment, and de novo antibody generation within unified computational workflows. Continued advances in foundation models, multimodal learning, and automated experimental feedback are expected to further accelerate antibody discovery and contribute to the development of safer, more effective, and increasingly personalized therapeutic antibodies.

## Figures and Tables

**Table 1:** Major public resources supporting computational antibody engineering.

Database/Resource	Primary Data Type	Key Content	Main Applications in Antibody Engineering	Ref.
**Protein Data Bank (PDB)**	Experimentally determined 3D structures	Protein and antibody atomic structures, experimental coordinates, validation data	Structural modelling, docking, template selection, structural analysis	[48]
**SAbDab**	Antibody structures	Curated antibody structures with antigen information, chain pairing, affinity data, structural annotations	Antibody structure prediction, CDR analysis, benchmarking, structural datasets	[1]
**PyIgClassify**	CDR structural classifications	Canonical CDR conformations, IMGT germline assignments, structural clusters	CDR classification, structure prediction, computational antibody design	[2]
**AbDb**	Processed antibody structures	Pre-numbered Fv fragments, multiple numbering schemes (Kabat, Chothia, Martin), antigen annotations	Comparative structural analyses, standardized datasets	[4]
**Thera-SAbDab**	Therapeutic antibody structures	WHO-recognized therapeutic antibodies linked to experimentally solved structures	Therapeutic antibody engineering, structural coverage assessment, sequence comparison	[7]
**CoV-AbDab**	Coronavirus antibodies	Antibody sequences, germline assignments, epitopes, homology models, structural metadata	SARS-CoV-2 antibody discovery, comparative analysis, therapeutic development	[46]
**Observed Antibody Space (OAS)**	Antibody repertoire sequencing	IMGT-numbered antibody sequences with standardized metadata from Ig-seq studies	Repertoire mining, comparative immunogenomics, AI training datasets	[5]
**Pan Immune Repertoire Database (PIRD)**	TCR/BCR repertoires	Annotated immune repertoires, VDJ assignments, CDRs, metadata, visualization tools	Repertoire analysis, machine learning, comparative studies	[44]
**AB-Bind**	Mutational binding data	Experimentally measured ΔΔG values for antibody mutations	Benchmarking affinity prediction algorithms, model validation, antibody optimization	[3]
**Immune Epitope Database (IEDB)**	Functional immunological data	Antibody epitopes, T-cell epitopes, receptor sequences, structural links	Epitope analysis, antigen selection, vaccine and antibody development	[6]

**Table 2:** Representative protein language models for antibody engineering.

Model	Architecture/Training Strategy	Training Data	Output Representation	Representative Applications	Main Advantages	Ref.
ESM-1b	Transformer, masked language modeling (self-supervised)	~250 million protein sequences (UniParc)	Residue-level and sequence-level embeddings	Structure prediction, mutation effect prediction, protein function prediction, transfer learning	Captures evolutionary and structural information directly from sequence	[57]
ESM-2/ESMFold	Scaled Transformer language model	Large-scale protein sequence databases	High-dimensional embeddings with structural information	Atomic-level structure prediction, zero-shot prediction, feature extraction	Learns structural information without MSA or templates	[23]
ProtBERT	BERT-based Transformer	Billions of amino acids from UniRef	Context-aware sequence embeddings	Protein classification, transfer learning, downstream predictive tasks	General-purpose protein representations transferable across tasks	[64]
ProtGPT2	Autoregressive Transformer	Large protein sequence corpus	Generative sequence representations	De novo protein generation, sequence design	Generates novel protein sequences following natural sequence statistics	[64]
AbLang	Antibody-specific Transformer language model	Observed Antibody Space (OAS) database	Residue embeddings (res-codings), sequence embeddings (seq-codings), amino acid probabilities	Antibody completion, residue engineering, antibody property prediction	Learns antibody-specific sequence semantics and mutation patterns	[20]
DeepSequence	Bayesian variational autoencoder (deep latent-variable model)	Multiple sequence alignments of protein families	Latent sequence representations	Mutation effect prediction, exploration of sequence space	Captures higher-order sequence dependencies and epistatic interactions	[66]

**Table 3:** Comparison of artificial intelligence approaches for de novo antibody design.

Computational Approach	Representative Methods	Primary Objective	Key Advantages	Major Limitations	Representative References
**Autoregressive language models**	IgLM, NanoNet	Generate novel antibody sequences	Efficient sequence generation; no multiple sequence alignment required; controllable sequence infilling	No explicit structural optimization	[59,72]
**Protein language models**	ESM, AntiFold (PLM backbone)	Learn evolutionary representations for antibody design	Capture evolutionary constraints; support transfer learning and inverse folding	Structure predicted indirectly	[58,73]
**Graph neural networks (GNNs)**	RefineGNN, ProteinSolver	Joint sequence–structure optimization	Models residue interactions; iterative refinement of sequence and structure	Limited scalability for highly complex systems	[67,74]
**Variational autoencoders (VAEs)**	Ig-VAE	Generate novel antibody backbone conformations	Direct 3D backbone generation; exploration of structural space	Limited control over antigen specificity	[75]
**Diffusion models**	DiffAb, AbX	Joint generation of antibody sequence and structure	Antigen-conditioned design; diverse structural sampling; high-quality generation	High computational cost; requires structural data	[69,70]
**Preference-optimized diffusion models**	ABDPO	Improve antigen-specific binding through preference optimization	Simultaneous optimization of structure and predicted affinity	Relies on accurate energy estimation	[71]
**Full-atom generative models**	AbDiffuser, dyMEAN	Full-atom antibody sequence–structure co-design	High structural accuracy; realistic atomic interactions	Computationally intensive	[68,76]
**Inverse folding models**	AntiFold	Design sequences compatible with predefined structures	Maintains structural integrity; suitable for affinity maturation	Requires an existing backbone structure	[73]
**Dynamic antigen-aware models**	dyAb	Design antibodies against flexible antigens	Models antigen conformational changes; improved biological realism	Increased model complexity	[77]
**RFdiffusion-based design**	RFdiffusion	Atomically accurate de novo antibody generation	Epitope-specific design; experimental validation; VHH, scFv and IgG generation	Low experimental hit rate; affinity maturation often required	[78,79]

**Table 4:** Evolution of computational antibody engineering: from experimental discovery to generative artificial intelligence.

Era	Dominant Paradigm	Key Technological Advances	Main Achievements	Remaining Limitations	Representative References
**Experimental antibody engineering**	Experimental selection	Hybridoma technology, phage display, combinatorial libraries, affinity maturation	Isolation of antigen-specific antibodies and establishment of antibody repertoires	Labor-intensive discovery and limited throughput	[24,25,29,30,35]
**Structure-based engineering**	Physics-based modeling	Rosetta, molecular docking, molecular dynamics, free-energy calculations	Rational optimization of antibody affinity and stability	Requires experimentally determined structures and extensive computational resources	[12,13,56]
**Machine learning**	Feature-based prediction	Supervised learning using engineered sequence and structural descriptors	Prediction of affinity, stability and developability	Performance depends on handcrafted features and training datasets	[11,15,16,19,20]
**Deep learning**	Representation learning	CNNs, GNNs and transformer-based structure prediction	Accurate prediction of antibody structure and functional properties	Limited interpretability and dependence on large annotated datasets	[14,17,23]
**Protein language models**	Self-supervised learning	ESM, ProtBERT, AntiBERTa and AbLang	Context-aware sequence representations and transfer learning	High computational cost and limited biological interpretability	[57,58,60,63,64]
**Generative artificial intelligence**	De novo antibody design	Diffusion models, autoregressive models and foundation models	Simultaneous generation and optimization of antibody sequences and structures	Experimental validation and clinical translation remain essential	[67,68,70,75,90]

**Table 5:** Comparative overview of representative AI models.

Model	AI Approach	Primary Application	Distinctive Feature	Ref.
**DeepAb**	Interpretable deep learning	Antibody structure prediction	Predicts inter-residue geometries to reconstruct antibody structures	[22]
**ABlooper**	Equivariant deep learning (GNN)	CDR loop prediction	End-to-end prediction of antibody CDR loops with confidence estimation	[18]
**AlphaFold**	Deep learning	Protein structure prediction	Near-experimental accuracy for protein structure prediction	[15]
**IgFold**	Language model + graph neural network	Antibody structure prediction	Fast antibody-specific structure prediction directly from sequence	[22]
**ESMFold**	Protein language model	Protein structure prediction	Direct atomic-level structure prediction from sequence without MSA	[23]
**AbLang**	Antibody language model	Antibody sequence modelling	Antibody-specific language model for sequence completion and representation	[20]
**DiffAb**	Diffusion model	Antibody sequence–structure co-design	Antigen-conditioned joint sequence and structure generation	[67]
**AbDiffuser**	Equivariant physics-informed diffusion model	Full-atom antibody generation	Joint generation of full-atom antibody structures and sequences with experimental validation	[68]
**AbX**	Score-based diffusion model	Antigen-specific antibody design	Integrates evolutionary, physical and geometric constraints into antibody generation	[70]
**ABDPO**	Preference optimization of diffusion models	Antibody optimization	Energy-based preference optimization for generating antibodies with improved binding affinity	[71]
**RFdiffusion**	Diffusion model	De novo protein and antibody design	Structure-guided generative design of proteins and antibodies from user-defined specifications	[78,79]
**ProteinMPNN**	Inverse folding/message-passing neural network	Structure-conditioned protein sequence design	Generates amino acid sequences compatible with a predefined protein backbone; serves as a foundation for antibody-specific models such as AbMPNN	[99,100]
**BoltzGen**	Unified all-atom diffusion/sequence–structure co-design	Antigen-conditioned binder and nanobody design	Jointly performs structure prediction and binder design, enabling simultaneous generation of sequence and structure with optional binding-site constraints	[101,102]

## Data Availability

No new data were created or analyzed in this study. Data sharing is not applicable to this article.

## Abbreviations

The following abbreviations are used in this manuscript:

AB-Bind	Antibody Binding Affinity Database
ABDPO	Antibody Direct Preference Optimization
AbDb	Antibody Database
AbDiffuser	Antibody Diffusion Model
AbLang	Antibody Language Model
ABlooper	Antibody Loop Structure Prediction Model
AbX	Antibody X
AHo	Honegger Antibody Numbering Scheme
AI	Artificial Intelligence
AntiBERTa	Antibody Bidirectional Encoder Representations from Transformers
AntiFold	Antibody Sequence Design Model
AlphaFold	AlphaFold Protein Structure Prediction System
AlphaFold-Multimer	AlphaFold Multimer Structure Prediction System
BCR	B-Cell Receptor
CDR	Complementarity-Determining Region
CDR-H3	Complementarity-Determining Region of the Heavy Chain 3
CoV-AbDab	Coronavirus Antibody Database
DeepAb	Deep Learning-Based Antibody Structure Prediction Model
ESM	Evolutionary Scale Modeling
ESMFold	Evolutionary Scale Modeling Fold
Fc	Fragment Crystallizable
Fv	Fragment Variable
GNN	Graph Neural Network
H1–H3	Heavy-Chain Complementarity-Determining Regions 1–3
HuCAL	Human Combinatorial Antibody Library
IEDB	Immune Epitope Database
Ig	Immunoglobulin
IgFold	Immunoglobulin Fold
IMGT	International ImMunoGeneTics Information System
L1–L3	Light-Chain Complementarity-Determining Regions 1–3
MD	Molecular Dynamics
MHC	Major Histocompatibility Complex
MiXCR	MiXCR Immune Repertoire Analysis Software
MSA	Multiple Sequence Alignment
NGS	Next-Generation Sequencing
NLP	Natural Language Processing
OAS	Observed Antibody Space
PDB	Protein Data Bank
PCR	Polymerase Chain Reaction
PIRD	Pan Immune Repertoire Database
PLM	Protein Language Model
ProtBERT	Protein Bidirectional Encoder Representations from Transformers
ProtGPT2	Protein Generative Pre-trained Transformer 2
RFdiffusion	RoseTTAFold Diffusion Model
RFdiffusion-antibody	RFdiffusion for Antibody Design
RNA	Ribonucleic Acid
Rosetta	Rosetta Macromolecular Modeling Suite
SAbDab	Structural Antibody Database
SARS-CoV-2	Severe Acute Respiratory Syndrome Coronavirus 2
SVM	Support Vector Machine
TCR	T-Cell Receptor
UniParc	Universal Protein Archive
UniRef	UniProt Reference Clusters
V(D)J	Variable, Diversity, and Joining Gene Segments
VH	Variable Heavy Chain
VL	Variable Light Chain
WHO	World Health Organization
ΔΔG	Change in Gibbs Free Energy upon Mutation
